# Year in Review: Axial Spondyloarthritis

**DOI:** 10.31138/mjr.300925.era

**Published:** 2026-01-08

**Authors:** Dimitrios Katsifis-Nezis, Antonis Fanouriakis

**Affiliations:** Rheumatology and Clinical Immunology, Attikon University Hospital, Medical School National and Kapodistrian, University of Athens, Greece

**Keywords:** axial spondyloarthritis, review, delayed diagnosis, management, MRI, AI

## Abstract

Axial spondyloarthritis is a chronic inflammatory disease primarily affecting the sacroiliac joints and spine. Over the past two years, significant progress has been made across multiple domains of spondyloarthritis research. The current review critically summarises key advancements in research during 2024–2025, covering pathophysiology, epidemiology, diagnosis, imaging, and clinical management. Notable findings include novel immune stromal interactions, such as IL-17 extracellular vesicles, IL-33 induced TREM2^+^ macrophages and disease specific CD8^+^ T cell responses that provide insight into bone remodelling and inflammation. Epidemiological data confirm persistent diagnostic delays, though targeted referral strategies show promise. Advances in imaging include an international consensus on MRI protocols and the growing use of ultrasound for the assessment of enthesitis. Artificial intelligence models demonstrate strong performance in early diagnosis, imaging interpretation and disease activity prediction and prognosis, while advances in therapy include the first head-to-head trial and numerous real-world data.

## INTRODUCTION

Axial spondyloarthritis (axSpA) is a chronic inflammatory disease that primarily affects the sacroiliac joints (SIJ) and spine, but occasionally extends to peripheral joints or involves extra-articular sites, such as the eyes, skin, and gastrointestinal system.^1^ In this review, we highlight major research articles published between 2024 and 2025, with a focus on epidemiology, novel imaging modalities and evolving treatment paradigms that are reshaping the care of patients with this disease.

## METHODS

A comprehensive literature search was conducted using the PubMed database and Google Scholar search engine to identify relevant publications from January 2024 to August 2025. The search strategy combined terms and keywords, including “axial spondyloarthritis”, “ankylosing spondylitis”, “early diagnosis”, “ epidemiology”, “ pathophysiology”, “magnetic resonance imaging”, “tumor necrosis factor inhibitors”, “interleukin-17 inhibitors”, “Janus kinase inhibitors”, “machine learning” and “ artificial intelligence”. Searches were limited to English language, peer reviewed studies conducted in humans.

To ensure thoroughness, the reference lists of included papers were manually screened, along with recent abstracts presented at EULAR 2024–2025 and ACR 2024–2025. Major rheumatology journals such as Annals of the Rheumatic Diseases and Arthritis & Rheumatology were systematically reviewed for additional relevant studies published within the same period. Supplementary studies were identified through snowballing of reference lists. Study selection was performed at the authors’ discretion, considering relevance, quality, and impact on the advancement of the field.

## PATHOPHYSIOLOGY

### Bone remodelling and new bone formation

Pathological new bone formation, manifesting as syndesmophytes and eventual ankylosis, is a hallmark feature of axSpA. In 2024, Feng et al. used single cell RNA sequencing from spinal entheses derived from both patients and healthy controls. They reported that early stage CD99_G1 neutrophils appear to interact closely with adipo–CXCL12 abundant reticular cells in axSpA, and express a repertoire of secreted factors with pro-osteogenic potential.^2^ In parallel, Wang et al. demonstrated that ligament derived extracellular vesicles containing interleukin (IL)-17A may also contribute to aberrant bone formation, while another study identified IL-33-induced TREM2^+^ macrophages as key players in coupling inflammation to pathological bone formation.^3,4^

#### CD8^+^ T cells

Although axSpA is traditionally considered to be closer to autoinflammation rather than autoimmunity due to its lack of autoantibodies, adaptive immunity has also been implicated in its pathogenesis. Guan and colleagues employed a spontaneous radiographic axSpA (r-axSpA) cynomolgus monkey model, to find notably higher numbers of CD8^+^ T cells compared to controls in the vertebral bone marrow and intervertebral disc lesions, alongside increased CD4^+^ T cells and CD11b^+^ cells. The expansion of CD8^+^ T cells suggests they may recognise antigens presented by MHC class I molecules, pointing to a cytotoxic immune process that could contribute to axSpA pathogenesis.^5^

#### Microbiome

Subclinical gastrointestinal inflammation is present in up to 50% of patients with axSpA and 5–10% eventually develop inflammatory bowel disease, pointing to a gut– joint axis.^6^ A multi-omics approach of gut microbiome confirmed that patients with axSpA have altered microbial diversity and bacterial phylum distribution when compared with healthy controls,^7^ a finding consistent with previous reports.^8^ Interestingly, treating axSpA patients with TNF inhibitors (TNFi) partially restored the gut microbiome and metabolomic profile closer to that of healthy controls.

Altogether, these findings improve our understanding of axSpA pathogenesis and highlight potential therapeutic targets, beyond conventional cytokine blockade.

## EPIDEMIOLOGY

### Diagnostic delay and associated burden

Despite increasing clinical awareness, axSpA remains frequently underdiagnosed and often identified late in disease course. Recent studies confirm that diagnostic delay continues to be a major issue globally, with important implications for disease outcomes and healthcare burden.

A multinational survey published in 2025 by Poddubnyy et al. evaluated the diagnostic delay in axSpA across diverse populations. Drawing from over 5,300 patient-reported cases across 27 countries, the study found a mean diagnostic delay of 7.4 years (median 4.0), with substantial variation by region, ranging from 4.2 years in Asia to over 10 years in South Africa. Factors independently associated with longer diagnostic delay included younger age at symptom onset, female sex, a higher number of healthcare providers seen prior to diagnosis and a history of uveitis.^9^ In a separate observational cohort involving 404 participants, Ferrandiz-Espadin et al. evaluated the impact of social determinants of health on diagnostic delay in r-axSpA. The findings indicated that patients with lower socioeconomic status, as assessed by the THRIVE questionnaire (which addresses housing stability, food security, transportation access, medication affordability, employment, education, and caregiver status), experienced a 21% longer time from the onset of back pain to diagnosis.^10^ Taken together, these studies indicate that diagnostic delay is not solely a matter of clinical awareness, but may also be a reflection of societal parameters.

Contrary to these patterns, a nationwide, population-based study revealed a consistent increase in the prevalence of axSpA among draft-eligible military populations in South Korea between 2014 and 2023, primarily driven by a rise in r-axSpA.^11^ This trend suggests improved detection, not necessarily an increase in true disease burden. Therefore, while underdiagnosis remains an issue, early identification of axSpA may be improving in certain populations, particularly in places where well-structured health screening programs are in operation.

Supporting this positive trend, Perez-Alamino et al., using data from the international ASAS-COMOSPA study, reported a marked reduction in diagnostic delay for spondyloarthritis, with the median time to diagnosis decreasing from 19 years before 1980 to 0.4 years after 2010. The proportion of patients diagnosed within two years rose from 15% to 88%, being more likely in those with axial or peripheral symptoms and less likely in women or patients presenting with isolated extra-musculoskeletal features.^12^ Therefore, while regional differences do exist, long-term trends suggest a broader shift in global diagnostic practice.

Apart from its clinical consequences, delayed diagnosis of axSpA imposes a significant economic burden. In the UK, Zanghelini et al. estimated that diagnostic delays could cost up to £193,512 per patient, with total national costs ranging from £3.1 to £12.5 billion annually, depending on disease prevalence.^13^ Similarly, in Spain, the mean total healthcare cost per patient accumulated over 3 years was found to be €25,812.6, 57% higher in patients with a delayed diagnosis, compared to patients with earlier diagnosis (<5.4 years).^14^ In addition, real-world data from Poland demonstrated a substantial work productivity loss among patients with axSpA, comparable to that seen in RA and PsA.^15^ Adding to the complexity, multicentre data comparing RA, PsA, and axSpA found hypertension, dyslipidaemia, and obesity most common. AxSpA had a lower overall burden compared to other rheumatic diseases but remained at risk for osteoporosis and structural damage.^16^

## DIAGNOSIS

### Classification and Terminology

In 2024, the Assessment of SpondyloArthritis International Society (ASAS) reached consensus on revisiting the nomenclature of axSpA. Experts agreed that ax-SpA should be the overarching term, while distinctions, such as r-axSpA versus non-radiographic- (nr-) axSpA remain relevant mainly for research purposes. Another important decision was that the terms ankylosing spondylitis (AS) and r-axSpA can be used interchangeably, although r-axSpA is currently the preferred term for the sake of consistency.^17^

Consensus was also achieved within ASAS for the definition of early axSpA as axial symptom duration ≤2 years, regardless of the presence or absence of radiographic damage.^18^ Adopting this standardised definition would ensure greater consistency in early-stage axSpA research.

### Initiatives for Early Recognition

While terminology evolves at the classification level, recent efforts have also emphasised on practical strategies for earlier clinical recognition.

The SHERPAS study examined the feasibility of identifying axSpA among young adults with chronic back pain (CBP) referred to non-rheumatology specialists. Using a self-administered questionnaire focused on back pain characteristics and SpA features, along with spinal and SIJ MRI, approximately 8% of referred individuals were ultimately diagnosed with definite or probable axSpA. Evaluation by a rheumatologist served as the diagnostic gold standard. Buttock pain, shorter duration of symptoms, increased CRP, HLA-B27 positivity and good response to nonsteroidal anti-inflammatory drugs (NSAIDS) were more common in axSpA patients. Active inflammatory lesions, as opposed to structural lesions, in MRI of the spine were more frequently found in the axSpA population. For the SIJ MRI, both active and structural lesions were more frequently found in axSpA patients.^19^ This study indicates that a notable minority of young adults with CBP seen outside rheumatology settings may actually have axSpA. Similarly, Maksymowych et al. in the Screening for Axial Spondyloarthritis in Psoriasis, Iritis, and Colitis (SASPIC) study, referred patients with undiagnosed chronic back pain from specialty clinics for rheumatologic evaluation. They demonstrated that approximately 30% of patients with psoriasis or inflammatory bowel disease and 60% of those with uveitis, who had chronic back pain were diagnosed with axSpA, reinforcing the importance of targeted triage in at-risk population.^20^ Together, these studies argue for the value of targeted triage strategies in both generalist and specialty clinics, where non-rheumatologists are often the first point of contact.

Focusing on early disease, Garcia-Salinas et al. found that approximately 38% of patients in the Argentinian Reuma-Check cohort met the definition of early axSpA, defined as symptom duration ≤2 years at diagnosis. Compared to those with more established disease, patients with early axSpA were less likely to smoke, had lower rates of radiographic sacroiliitis (37% vs. 71%) and more frequently reported a family history of SpA (38% vs. 23%).^21^ Although the data are specific to a Latin American population and may not be broadly generalisable, the study contributes to mapping the clinical phenotype of early axSpA and, similarly to the SHERPAS study, underscores the importance of recognising distinct features in the early stages of the disease. Moreover, the SPACE cohort further explored whether early axSpA could be reliably diagnosed in the initial stages, and how the diagnosis evolves over time.^22^ The study enrolled 555 individuals aged below 45 years old with CBP lasting less than two years. The patients underwent a thorough diagnostic work-up, including MRI, and rheumatologists made a diagnosis of axSpA or non-axSpA with an accompanying confidence score (0–10). Two years later, diagnoses were re-evaluated. At baseline, one third of patients (32%) were diagnosed with definite axSpA (level of confidence ≥ 7) and this diagnosis remained stable at the two-year interval (30%), signifying a strong initial specificity. The diagnosis was revised in only 5% of patients, while 8% received a diagnosis during follow-up. Of those, a good response to NSAIDS and MRI sacroiliitis in MRI were more frequently observed, while male sex and HLA-B27 positivity were more prevalent in the MRI-sacroiliitis cases. Of note, diagnostic uncertainty remained in 30% of cases after 2 years. One limitation was the 29% loss to follow-up, and that the findings largely reflected academic centre practices. Still, this was the first study to formally show that rheumatologists can reliably diagnose axSpA at first diagnosis (mean symptom duration 13 months). Repeating MRI assessments had limited impact overall, but can be helpful in HLA-B27–positive male patients with CBP. Overall, these findings argue for the early involvement of rheumatologists in axSpA diagnostic algorithms.

Furthermore, while global studies emphasise persistently delayed diagnosis, real-world initiatives demonstrate that change is possible. A 2024 UK-based service improvement project by Chan et al. achieved a dramatic reduction in mean diagnostic delay, from 9.8 years in 2008 to just 1.0 year in 2023, through integrated referral systems, public awareness campaigns, and primary care education. These findings indicate that structural reforms, rather than clinical awareness alone, are essential to improve early detection of axSpA. Such models, however, are the exception rather than the rule, and broader implementation remains an unmet need.^23^

Beyond individual screening initiatives, accurate diagnosis of axSpA relies on synthesising multiple domains of information rather than any single test in isolation. A recent meta-analysis assessed the diagnostic performance of clinical, laboratory, and imaging features of axSpA. Increased serum inflammatory markers, sacroiliitis in MRI and HLA-B27 status emerged as the most significant discriminators between axSpA and non-axSpA; HLA-B27 positivity was found less prevalent than expected. The presence of inflammatory back pain showed good negative predictive value, whereas extra-articular manifestations and peripheral arthritis provided further confirmation. Importantly, the positive likelihood ratios for most features, apart from imaging, were smaller than those reported in studies that informed the older Berlin diagnostic algorithm (a set of criteria combining clinical, laboratory, and imaging features to aid axSpA diagnosis).^24^ This raises the possibility that current diagnostic pathways may need updating and highlight the central role of imaging in raising or lowering diagnostic confidence, especially in early disease.^25^

## IMAGING

### Standardised MRI Acquisition Protocol & Imaging of the Spine

A major advance in 2024–2025 was the publication of an international consensus by ASAS and SPARTAN on the standardisation of MRI acquisition protocols for SIJ imaging.^26^ The expert panel recommended a minimum four-sequence protocol, consisting of three semi-coronal and one semi-axial sequence, including an inflammation-sensitive, a fat-sensitive and an erosion-sensitive sequence. All sequences should be oriented orthogonally to the sacrum, specifically using the dorsal cortex of the S2 vertebral body as a consistent anatomical reference. Though only focusing on image acquisition, the consensus addressed longstanding inconsistencies in MRI protocols and represents an important step toward the standardisation of imaging in axSpA.

Parallel advances were also made in imaging of the spine. Conventional radiography of the spine and the modified Stoke Ankylosing Spondylitis Spinal Score remain the only validated methods for assessing and quantifying changes in structural spinal lesions; however, they suffer from low sensitivity, as they typically detect change after a minimum of 2 years.^27^ A recent study introduced a novel MRI-based synthetic CT technique, which demonstrated markedly higher sensitivity than plain radiography, while maintaining very high specificity. Low-dose CT was used as the gold standard.^28^ This approach enables detailed structural damage assessment in the spine without exposing patients to ionising radiation, thus offers a promising tool for both clinical trials and longitudinal monitoring.

With homogenised acquisition protocols established and novel techniques emerging, recent data help clarify how MRI lesions must be interpreted across different disease patient populations.

### Age and Specificity of MRI Findings

Weber and colleagues investigated the effect of increasing age on active and structural MRI lesions in the SIJ among healthy volunteers and patients with non-specific back pain (NSBP) aged ≤45 years from three independent cohorts.^29^ They found that only a small proportion of healthy individuals and NSBP patients had erosions in ≥ 1 SIJ quadrant, with no clear increase in prevalence with age. None of the healthy individuals and only a minority of NSBP patients (2.3–4.3%) met the ≥ 3-quadrant cut-off indicative of ax-SpA, and no cases meeting this threshold occurred in the oldest age group. Fat metaplasia showed a slight age-related increase, though this was not consistent across cohorts. Bone marrow oedema was numerically more frequent in the oldest group of healthy individuals, whereas its prevalence did not increase with age in NSBP patients. These data demonstrate that structural lesions, especially erosions, remain highly specific for axSpA diagnosis and show no substantial increase with advancing age. These results contrast with older findings by Renson et al., who reported a high prevalence of structural lesions in healthy adults with increasing age.^30^ Weber et al. hypothesised that these findings may be a result of physiological SIJ contour variations classified as erosion. More studies are needed to elucidate this inconsistency between studies, which nevertheless highlight the importance of the standardised ASAS–SPARTAN MRI acquisition protocol in homogenising future cohorts.

Active lesions in MRI of the spine and SIJ are key features of diagnosis, especially in nr-axSpA. The observation by Torgutalp and colleagues that bone marrow oedema can occur in healthy individuals raises questions about the specificity of bone marrow oedema. In a large population-based MRI study of nearly 1,000 adults from the German National Cohort, active inflammatory changes were present in approximately 28.7% and structural changes in 24.0% of participants, yet changes compatible with axSpA were rare (0.5% and 1.1%, respectively).^31^ Older age, female sex, higher BMI, physically demanding work, and a history of pregnancy were linked to non-specific SIJ changes. These results confirm that various factors can contribute to MRI abnormalities, especially bone marrow oedema, underscoring the importance of evaluating both active and structural lesions in context, to avoid overdiagnosis of axSpA.

To add more complexity, in a real-world cross-sectional cohort study of older adults evaluated for CBP, Ziade et al. found significant overlap in inflammatory and degenerative imaging features among degenerative disc disease, diffuse idiopathic skeletal hyperostosis (DISH), and axSpA. Interestingly, fat metaplasia and erosions were absent in patients with DISH, suggesting that the concurrent presence of both inflammatory and structural changes was more indicative of axSpA.^32^ Nevertheless, this study serves as a reminder of the inherent complexity of real-world patients and emphasises the value of a holistic, “gestalt” approach to the diagnosis and management of axSpA.

### Diagnostic Performance of Combined Active and Structural Lesions

In order to improve diagnostic accuracy in early disease, recent work has also compared different MRI definitions to determine which criteria best balance sensitivity and specificity. Definitions for MRI positivity differ between the MRI Study Group and ASAS criteria (**Table 1**)^33,34^; the former incorporates both active and structural lesions for the diagnosis, while the latter only active lesions. A 2024 prospective study evaluated these two approaches in patients with suspected early axSpA from the SPACE cohort.^35^ The ASAS definition of active lesions showed the best sensitivity (40% vs 31%) with no decrease in specificity, when compared with MRI study Group (98% vs 99%). In patients with a short duration of symptoms, the addition of structural lesions offered only a limited improvement in the sensitivity for axSpA detection. Notably, the combination of the ASAS active lesion definition with structural lesion criteria provided the best performance. Overall, despite a more lenient approach in the definition of active lesions, the ASAS criteria showed higher sensitivity and specificity, suggesting the importance of inflammatory lesions in the diagnosis of early axSpA in young patients; structural lesions have complementary, yet limited value in early disease. It is important to emphasise, however, that overall sensitivity of both definitions remains modest and is not sufficient to establish the diagnosis with certainty.

**Table 1.** MRI Definitions (comparison of ASAS-MRI-SIJ+ and MRI Study Group criteria).

**Feature**	**ASAS-MRI SIJ+**	**MRI Study Group**
Primary focus	Active lesions	Active & Structural lesions
Active lesion definition	BME clearly present on ≥2 consecutive slices in typical locations	BME in ≥4 SIJ quadrants or same location across ≥3 consecutive slices
Structural lesion definition	Not included	Erosions in ≥3 quadrants, fat lesions in ≥5 quadrants, erosions in same location across ≥2 slices, fat lesions in ≥3 consecutive slices, or deep fat lesion (>1 cm)

ASAS: Assessment of Spondyloarthritis International Society; MRI: magnetic resonance imaging; SIJ: sacroiliac joint; BME: bone marrow oedema.

### Radiographic Progression: Evidence from the DESIR inception cohort

Long-term data on radiographic progression further complement these diagnostic insights. Through a 10-year follow-up of patients with recent onset axSpA (<3 years), Molto and colleagues confirmed the low radiographic progression from nr-axSpA to r-axSpA. Less than 10% of the cohort showed radiographic progression. When accounting for TNF inhibitor (TNFi) use, progression was even lower (<5%), indicating the potential protective effect of TNFi therapy. Baseline BME in SIJ especially in HLA-B27 positive patients served as the strongest risk factor.^36^

### Ultrasonography

While MRI continues to dominate imaging research, 2024–2025 also saw progress in ultrasound, particularly in efforts to standardise the assessment of enthesitis.

In everyday practice, clinical assessment of enthesitis is often confounded by factors, such as fibromyalgia and coexisting degenerative changes, which may mimic or obscure true inflammatory involvement.^37^ Di Matteo et al. evaluated the level of agreement between ultrasound-detected and clinically-detected enthesitis, and estimated the prevalence of subclinical enthesitis and its significance in patients with axSpA. Their results showed that clinical enthesitis was found in more than one-third (38.3%) of patients. Notably, the majority of these cases (68.4%) were not confirmed by ultrasound. On the contrary, 15% of the population demonstrated enthesitis on ultrasound, despite the absence of clinical findings, and enthesitis was clinically significant, because it was associated with local structural damage.^38^ Therefore, the presence of enthesitis can be overestimated through clinical examination in patients with axSpA, and the authors propose the need for a composite diagnostic tool that combines clinical and ultrasound data to improve accuracy.

Furthermore, in a recent multicentre study, power Doppler signal at the enthesis and bone erosions emerged as the ultrasound features with the greatest ability to distinguish SpA from other conditions. The Achilles tendon enthesis proved to be the most diagnostically informative site, lending support to a hierarchical, site-focused strategy when performing ultrasound assessments.^39^ Taking all these into account, Di Matteo and colleagues developed the DEUS (Defining Enthesitis on Ultrasound in Spondyloarthritis) algorithm, which incorporates both inflammatory and structural sonographic features alongside clinical data, to stand-ardise enthesitis assessment. This framework aims to enhance diagnostic precision, reduce interobserver variability, and help differentiate SpA-related enthesitis from mechanical or degenerative changes in routine clinical practice.^40^

## ARTIFICIAL INTELLIGENCE

Artificial intelligence (AI) has rapidly emerged as a transformative tool in axSpA research and care, with publications in 2024–2025 reflecting advances in both early diagnosis and risk assessment, as well as personalised treatment.

### Diagnosis

Two recent studies demonstrated the potential of AI for early diagnosis of axSpA. Redeker and colleagues developed a diagnostic model using machine-learning, based on retrospectively retrieved clinical, laboratory and imaging data of patients with CBP of unknown cause. The imaging component was derived from radiologists’ reports, rather than direct image analysis. The model showed high discriminatory ability in distinguishing axSpA from non-axSpA patients, achieving an overall accuracy of 89% (sensitivity 95% - specificity 75%). HLA-B27 positivity, insidious onset of low back pain, presence of erosions in the sacroiliac joint and elevated CRP were the most influential predictors driving model performance.^41^ Notably, this AI approach outperformed the ASAS-adopted Berlin algorithm in diagnostic accuracy.^42^ Similarly, Xie et al. used a deep learning approach to SIJ MRI and combined it with clinical variables. Clinical risk factors identified were age, sex, and HLA-B27 status. Their model also outperformed the ASAS clinical criteria in both sensitivity and specificity.^43,44^

Given that diagnostic delay remains a major challenge in axSpA, these AI-driven strategies could in the future facilitate earlier recognition and targeted referral, particularly when patients initially present in non-rheumatology settings.

### Imaging Analysis

Imaging retains a central role in the diagnosis of ax-SpA. Recent literature shows that the integration of AI in medical imaging can enhance its diagnostic and prognostic value. In X-ray studies, Meng et al. developed and externally validated a deep learning model that achieved 90.1% accuracy for detecting radiographic sacroiliitis and 63.9% accuracy for grading according to the modified New York criteria. Beyond its stand-alone diagnostic performance, the model improved junior radiologists’ accuracy from 91% to 97% for detection and from 75% to 89% for grading, pointing to its potential as a performance-enhancing tool in clinical practice.^45^

**Table 2.** Key publications in spondyloarthritis (2024–2025).

**Domain**	**Year (Reference)**	**Key Finding**
**Pathophysiology**	2024 (Feng et al.)^2^	CD99^+^G1 neutrophils interacting with stromal cells to promote osteogenesis.
	2024 (Wang et al.)^3^	IL-17A containing extracellular vesicles promote osteogenesis.
	2025 (Hao et al.)^4^	IL-33–induced TREM2^+^ macrophages couple inflammation to pathological new bone formation.
	2024 (Guan et al.)^5^	Expanded CD8^+^ T-cell populations suggest antigen driven cytotoxicity in axSpA lesions.
	2024 (Lai et al.)^7^	Gut microbiome dysbiosis partially normalised following TNF inhibition.
**Epidemiology**	2025 (Poddubnyy et al.)^9^	Global diagnostic delay averages 7.4 years with pronounced regional variation.
	2025 (Perez-Alamino et al.)^12^	Diagnostic delay has shortened markedly over recent decades.
	2025 (Ferrandiz-Espadin et al.)^10^	Socioeconomic disparities contribute to prolonged time to diagnosis.
	2025 (Zanghelini et al.; Berbel-Arcobé et al.)^13,14^	Delayed diagnosis confers substantial economic burden.
**Diagnosis & Early Recognition**	2024 (Navarro-Compán et al.)^18^	ASAS consensus defines early axSpA as ≤2 years of axial symptoms.
	2025 (Benavent et al.)^19^	SHERPAS: 8% of young adults with chronic back pain had axSpA.
	2025 (Maksymowych et al.)^20^	30% of patients with psoriasis or inflammatory bowel disease and 60% of those with uveitis, who had chronic back pain were diagnosed with axSpA.
**Imaging (MRI & CT)**	2024 (Lambert et al.)^26^	ASAS–SPARTAN consensus standardised MRI acquisition for SI joints.
	2025 (Weber et al.)^29^	Age has minimal effect on structural SIJ lesions; erosions remain highly specific.
	2024 (de Bruin et al.)^35^	Combination of the ASAS active lesion definition with structural lesion criteria provided the best performance.
	2024 (Molto et al.)^36^	DESIR 10-year follow-up shows minimal radiographic progression.
**Ultrasound**	2025 (Di Matteo et al.)^38^	Clinical enthesitis is frequently overestimated; ultrasound detects subclinical lesions.
	2024 (Di Matteo et al.)^39^	Power Doppler and erosions are the most discriminative sonographic features.
	2025 (Di Matteo et al.)^40^	DEUS Index integrates clinical and ultrasound domains for standardised scoring.
**Artificial Intelligence**	2024 (Redeker et al.)^41^	Machine-learning diagnostic model achieved 89% accuracy for axSpA.
	2025 (Xie et al.)^43^	Deep learning on SIJ MRI outperformed ASAS clinical criteria.
	2024–2025 (Pradip et al.; Li et al.)^50,51^	AI models accurately predicted flares and uveitis risk.
**Therapy / Management**	2025 (Poddubnyy et al.)^54^	ASAS definition of “difficult-to-manage” and “treatment-refractory” axSpA proposed.
	2024 (Rudwaleit et al.)^55^	Long-term certolizumab pegol efficacy sustained across inflammatory subgroups.
	2025 (Hellamand et al.)^57^	Women reported poorer TNFi outcomes; phenotype and comorbidities contributed.
	2024 (Proft et al.)^61^	CONSUL: NSAID + TNFi combination not superior to TNFi alone.
	2024 (Baraliakos et al.)^75^	SURPASS: Secukinumab vs adalimumab: minimal radiographic progression in both arms.

Beyond diagnosis, AI models are now being applied to predict structural outcomes. Dorfner and colleagues, by using an anatomy-centred deep learning approach, found improved generalisability across different cohorts and the ability to predict 2-year radiographic progression.^46^ Similar results were shown for radiographic progression in the spine of patients with axSpA.^47^

Regarding MRI, Nicolaes et al. validated a previously trained deep-learning algorithm to identify SIJ inflammation. The algorithm showed moderate agreement with expert radiologists (74%) and acceptable performance in identifying inflammation in both radiographic and non-radiographic axSpA, in line with the 2009 ASAS MRI criteria.^48,49^ However, the study did not assess structural SIJ lesions and included only patients with an established axSpA diagnosis. While these findings underscore the potential of AI in MRI interpretation, broader validation across different populations and disease stages remains essential. Collectively, pending improvement of their performance, these algorithms cannot be considered at present suitable for clinical use.

### Prognosis & Outcome Prediction

In the area of predictive modeling, Pradip et al. used long short-term memory networks and neural networks to develop a model that forecasts flares in patients with axSpA. The model demonstrated excellent accuracy with high sensitivity and specificity (>89%) in predicting flares over a two-month period.50 Similarly, Li et al. used a machine learning approach in a cohort of 1508 axSpA patients to predict the onset of anterior uveitis, and they found a high predictive accuracy (92%) of their model. Secondary analyses identified hip involvement, smoking history and NSAID use as the key risk factors.^51^

## MANAGEMENT

The last two years have marked a period of significant advances in axSpA therapy, including the first head-to-head trial, real-world data, and omics-based studies. With current therapies, a substantial proportion of patients remain inadequately controlled, even after exposure to multiple biologics or targeted synthetic disease modifying antirheumatic drugs (b/tsDMARDs). Data from a nationwide Greek cohort suggest that about 10% of patients can be classified as difficult to treat, and up to 35% fail to reach treatment targets.^52,53^ To address this unmet need, in 2024 an ASAS task force proposed a consensus definition of Difficult-to-Manage (D2M) axSpA; patients who fail at least two biologic disease modifying antirheumatic drugs (bDMARDs) with different mechanisms of action and continue to exhibit uncontrolled signs and symptoms, perceived as problematic by either the physician or the patient, are categorised as D2M. A subgroup, termed treatment-refractory axSpA, is defined when alternative explanations are excluded and objective inflammation persists, indicated by ASDAS >2.1 together with elevated CRP or active sacroiliitis on MRI.^54^ This new definition serves as a basis through which to interpret therapeutic outcomes across different drug classes.

### TNF inhibition

Among TNFi, certolizumab pegol remains the only agent with newly longitudinal published data in axSpA. Rudwaleit et al conducted a post hoc analysis of the C-axSpAnd trial in patients with active nr-axSpA, assessing whether the long-term efficacy of certolizumab pegol was influenced by baseline factors, such as sacroiliitis on MRI and elevated serum CRP.^55^ Consistent with prior 1-year findings,^56^ clinical improvements were sustained through 3 years in patients with both MRI and CRP positivity or at least one of these inflammatory features. These findings validate the long-term efficacy of TNFi in nr-axSpA, but also suggest their applicability across all inflammatory subgroups.

In a large European real-world study of axSpA patients initiating TNFi therapy, women reported worse patient-reported outcomes than men. These gaps became more pronounced by 6 months and then remained stable through 2 years.^57^ These findings provide insight into the inconsistencies in the literature regarding TNFi efficacy across sexes.^58–60^ The authors attributed these sex differences partially to a distinct disease phenotype in women, with more peripheral arthritis and enthesitis and typically lower CRP levels, while confounders such as fibromyalgia and depression could also have contributed. Recognising this gap is essential to avoid underestimating disease activity and may guide more personalised treatment decisions in real-world practice.

In addition to longitudinal and real-world data, strategies to optimise TNFi efficacy have also been explored. In patients with r-axSpA at high risk of radiographic progression, the CONSUL study investigated the effect of adding a NSAID (celecoxib) to a TNFi (golimumab). Over two years, the combination did not achieve statistically significant superiority over golimumab monotherapy in preventing spinal radiographic progression.^61^ Nonetheless, a numerical trend in favour of combination therapy was noted, which the authors suggested might have clinical value in selected high-risk patients with residual disease activity despite bDMARD therapy. Although the study was designed with adequate statistical power based on historical progression rates, the relatively small sample size per treatment arm indicates that larger studies may be required to confirm these findings. Nevertheless, it provides further support for the effectiveness of TNFi in radiographic progression.

Lastly, previous research has shown that approximately 40% of patients do not respond adequately to TNFi.^62^ Talukdar et al. sought to identify baseline immunophenotypic signatures that might predict non-response in axSpA. In a small cohort of biologic-naïve male patients, they applied peripheral blood mononuclear cell transcriptomics and found that non-responders had a relative enrichment of monocytes, reduced T-cell populations, and upregulation of interferon-regulated genes.^63^ Although the sample size was limited and the results require validation in larger cohorts, this study represents an early step toward a personalised medicine approach in axSpA. These biomarkers, however, remain exploratory and far from clinical application.

### IL-17 Inhibition

The IL-17 axis is central in the pathogenesis of axSpA. Following approval of secukinumab, a multicentre European observational study was the first to provide evidence confirming its efficacy in both r-axSpA and nr-axSpA in the real world.^64^ Similarly, Jensen and colleagues recently demonstrated real world effectiveness of ixekizumab, a humanised IgG4 monoclonal antibody also targeting IL-17A, in both subtypes of the disease.^65^ Interestingly, most patients were bio-experienced and had already failed multiple biologics.

The newest IL-17 pathway biologic, bimekizumab, a humanised immunoglobulin G1 kappa monoclonal antibody that neutralises IL-17A, IL-17F, was approved after the BE MOBILE 1 & 2 studies.^66^ In both trials, bimekizumab reduced disease activity, enhanced patient-reported outcomes and lowered inflammatory markers, such as SIJ bone marrow oedema and serum CRP. These responses were maintained through a 2-year period, with approximately 60% achieving ASDAS low disease activity and more than 30% reaching inactive disease. Adverse events were consistent with prior reports, with COVID-19, nasopharyngitis and mild oral candidiasis being the most frequent.^67^ Importantly, minimal progression was also observed in the r-axSpA subpopulation,^68^ and low rates of uveitis were observed in both groups across pooled phase 2b/3 data.^69^

Finally, the new British Society for Rheumatology guidelines, published in 2025, state that inactive inflammatory bowel disease is not an absolute contraindication for IL-17 therapy, an important consideration to broaden therapeutic options for D2M patients.^70^

### JAK Inhibitors

Regarding JAK inhibitors (JAKi), longitudinal data from the SELECT-AXIS 2 study, comprising two parallel randomised, placebo-controlled trials, one in non-radiographic axSpA and one in radiographic axSpA with inadequate response to prior bDMARDs, have been published. In the r-axSpA (bDMARD failure) cohort, the 2-year open-label extension showed sustained efficacy, with >93% of patients having no spinal radiographic progression.^71^ To put in context, prior literature reports 79% non-progressors with secukinumab and 69.7% with infliximab over 2–4 years, though cross-trial comparisons should be made with caution due to differences in design and baseline risk.^72,73^ In the nr-axSpA sister trial, 1-year outcomes confirmed maintained efficacy compared to placebo.^74^

### Drug Class Comparisons

In 2024, the SURPASS study reported the first randomised, open-label, head-to-head trial in biologic-naïve patients with r-axSpA at high risk of structural progression, defined as high serum CRP and at least one syndesmophyte visible on radiograph. The trial compared secukinumab with a biosimilar adalimumab and found that spinal radiographic progression over two years was minimal in both groups, with no evidence of superiority for secukinumab over adalimumab.^75^ The study provides high-quality evidence supporting the effectiveness of both drugs in long-term structural outcomes.

Given the relatively high rates of TNFi failure real-world data have explored treatment sequencing strategies.^62^ Baraliakos and colleagues demonstrated that patients switching from a TNFi to upadacitinib achieved greater improvements in pain and joint involvement than those cycling to another TNFi or transitioning to an IL-17 inhibitor.^76^ A subsequent meta-analysis reinforced these findings, concluding that upadacitinib shows superior efficacy in both biologic-naïve and TNFi-experienced patients with active AS.^77^ Still, observational designs carry confounding-by-indication bias, and randomised head-to-head trials remain absent. In contrast, in biologic-naïve r-axSpA patients, a comparative study found tofacitinib, another JAKi, and adalimumab to have broadly similar effectiveness and safety outcomes.^78^

Although multiple IL-17 inhibitors are available, comparative trials are lacking. A meta-analysis suggested that bimekizumab may be more effective than secukinumab 150 mg, but not 300 mg, while differences with ixekizumab varied by population. However, the results are constrained by methodological limitations, including restricted baseline matching and reduced effective sample sizes, which may in turn limit the generalisability of the conclusions.^79^

In summary, despite considerable progress, most data remain indirect, post hoc, or extensions of randomised control trials. Moving forward, more head-to-head randomised trials, sex, and phenotype stratified analyses, as well as transcriptional studies in refractory disease are urgently needed.

## CONCLUSION

In 2024–2025, advances in pathophysiology identified new potential therapeutic targets, epidemiological data confirmed that diagnostic delay remains a global challenge despite regional progress, and standardised MRI protocols together with AI brought imaging closer to routine precision medicine. Real-world evidence, omics-based approaches, and the first head-to-head biologic trial expanded our understanding of drug efficacy in axSpA. Collectively, the past two years have brought progress across all aspects of axSpA research, generating data that lay the groundwork for more personalised diagnosis and management in the future.

Last but not the least, this review updates and broadens previous MJR discussions on axial spondyloarthritis, summarising recent advances of the field.
